# Supplementary material for: Comparative Analysis of Codon Usage Patterns in Nuclear and Chloroplast Genome of Dalbergia (Fabaceae)
Source: Genes (Basel). 2023 May 19;14(5):1110. doi: 10.3390/genes14051110 (PMC10217945; doi:10.3390/genes14051110)
Supplement: Supplementary file 1 [file genes-14-01110-s001.zip › Description of Additional Supplementary Files.pdf]

Description of Additional Supplementary Files

Supplementary Data 1

Description: Chloroplast genome of 25 *Dalbergia* species

Supplementary Data 2

Description: Amino acids of four types of synonymous codons

Supplementary Data 3

Description: Whole genome and chloroplast genome protein coding sequence information

Supplementary Data 4

Description: Codon usage indices of nuclear genome and chloroplast genome

Supplementary Data 5

Description: Codon GC content of 25 Dalbergia species

Supplementary Data 6

Description: Neutral plot analysis data for nuclear and chloroplast genomes

Supplementary Data 7

Description: Nuclear genome optimal codon

Supplementary Data 8

Description: Statistics of gene expression at different levels

Supplementary Data 9

Description: Information on the four bases of the nuclear genome in leaf tissues.

Supplementary Data 10

Description: Amino acid level gene expression information statistics
