# Supplementary material for: Comparative Analysis of Codon Usage Patterns in Nuclear and Chloroplast Genome of Dalbergia (Fabaceae)
Source: Genes (Basel). 2023 May 19;14(5):1110. doi: 10.3390/genes14051110 (PMC10217945; doi:10.3390/genes14051110)
Supplement: Supplementary file 1 [file genes-14-01110-s001.zip › Supplementary Information.pdf]

Supplementary Information

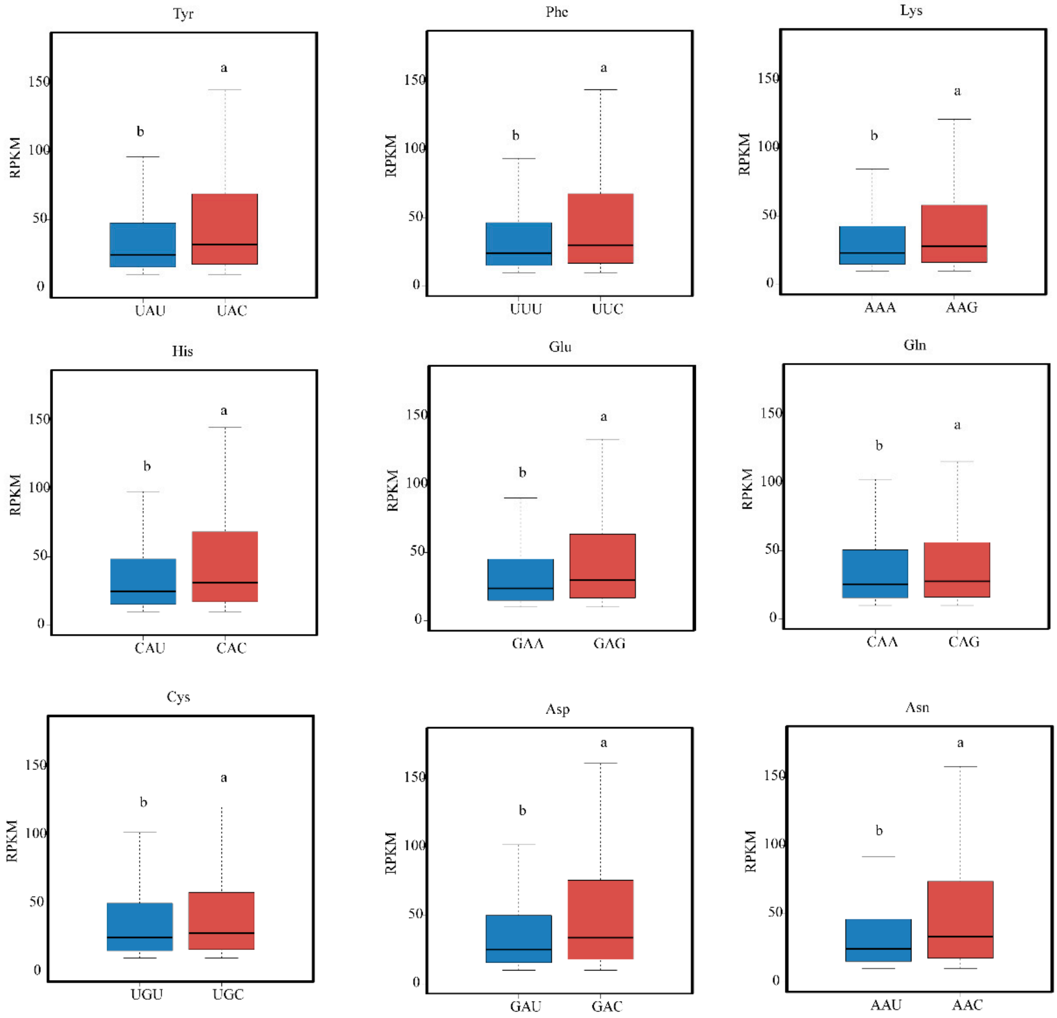

Figure S1. The relationship between RPKM value and synonymous codon preference in amino acid level (all amino acids with two synonymous codons, and lower a and b in boxplot meant the significant difference in statistical analysis)**.**

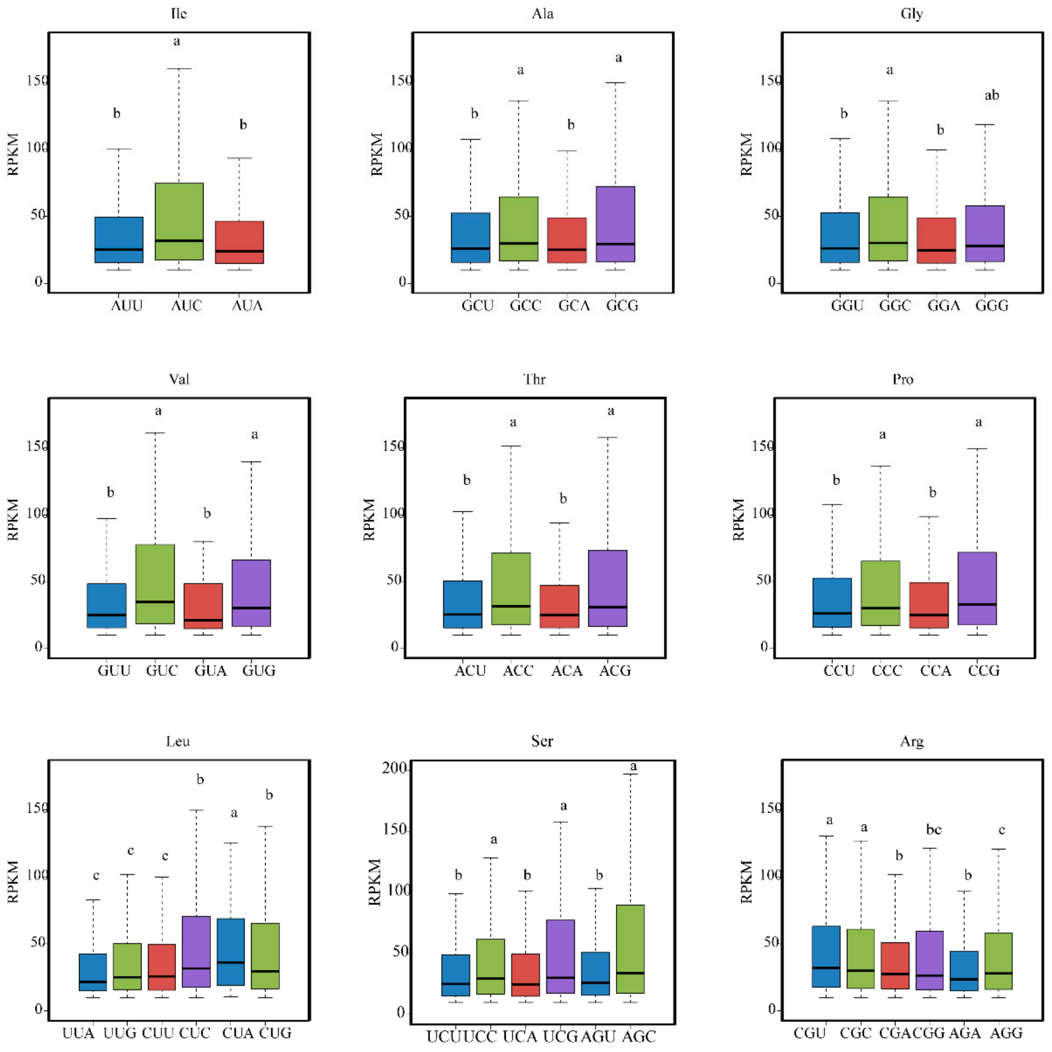

Figure S2. The relationship between RPKM value and synonymous codon preference in amino acid level (all amino acids with more than two synonymous codons, and lower a and b in boxplot meant the significant difference in statistical analysis).

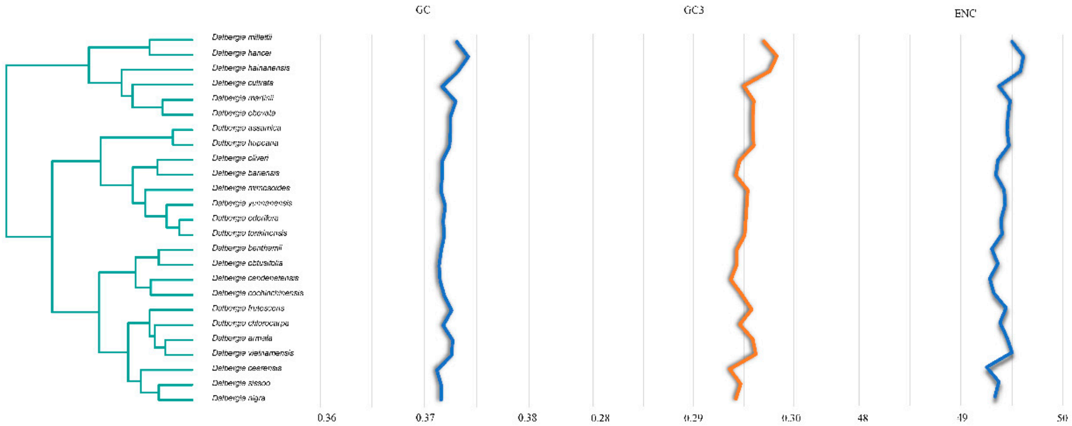

Figure S3. Clustering diagram based on RSCU values and its codon usage indicator

trend lines (GC content of chloroplast protein-coding sequences; GC3: content of G+C at the third position of chloroplast protein-coding sequences; ENC: number of effective codons).
